# Corticotropin-releasing factor system in the lateral septum: Implications in the pathophysiology of obesity

**DOI:** 10.3389/fnmol.2022.1020903

**Published:** 2022-09-20

**Authors:** Rossy Olivares-Barraza, José Luis Marcos, Jonathan Martínez-Pinto, Marco Fuenzalida, Javier A. Bravo, Katia Gysling, Ramón Sotomayor-Zárate

**Affiliations:** ^1^Facultad de Ciencias, Centro de Neurobiología y Fisiopatología Integrativa (CENFI), Instituto de Fisiología, Universidad de Valparaíso, Valparaíso, Chile; ^2^Programa de Doctorado en Ciencias Mención Neurociencias, Universidad de Valparaíso, Valparaíso, Chile; ^3^Programa de Doctorado en Ciencias e Ingeniería para la Salud, Universidad de Valparaíso, Valparaíso, Chile; ^4^Escuela de Ciencias Agrícolas y Veterinarias, Universidad Viña del Mar, Viña del Mar, Chile; ^5^Facultad de Ciencias, Grupo de NeuroGastroBioquímica, Instituto de Química, Pontificia Universidad Católica de Valparaíso, Valparaíso, Chile; ^6^Facultad de Ciencias Biológicas, Departmento de Biología Celular y Molecular, Pontificia Universidad Católica de Chile, Santiago, Chile

**Keywords:** obesity, feeding control, lateral septum (LS), CRF system, addiction

## Abstract

Obesity is a pandemic associated with lifestyles changes. These include excess intake of obesogenic foods and decreased physical activity. Brain areas, like the lateral hypothalamus (LH), ventral tegmental area (VTA), and nucleus accumbens (NAcc) have been linked in both homeostatic and hedonic control of feeding in experimental models of diet-induced obesity. Interestingly, these control systems are regulated by the lateral septum (LS), a relay of γ-aminobutyric (GABA) acid neurons (GABAergic neurons) that inhibit the LH and GABAergic interneurons of the VTA. Furthermore, the LS has a diverse receptor population for neurotransmitters and neuropeptides such as dopamine, glutamate, GABA and corticotropin-releasing factor (CRF), among others. Particularly, CRF a key player in the stress response, has been related to the development of overweight and obesity. Moreover, evidence shows that LS neurons neurophysiologically regulate reward and stress, although there is little evidence of LS taking part in homeostatic and hedonic feeding. In this review, we discuss the evidence that supports the role of LS and CRF on feeding, and how alterations in this system contribute to weight gain obesity.

## Introduction

Obesity is a global disease that has led the World Health Organization (WHO) to declare it a pandemic. Obesity is defined as an abnormal or excessive fat accumulation, which is detrimental to health, as there is an energy imbalance between calories consumed and calories expended. Quantitatively, the definition associated with the body mass index (BMI), which considers the height and weight of individuals, has defined the condition of obesity when the BMI is greater than 30 kg/m^2^ (World Health Organization [WHO], 2021). In addition, obesity has genetic and metabolic components, showing a higher prevalence in the offspring of obese parents, especially in developed societies (Cammarano et al., 2022). One of the most highly studied factors that lead to obesity is exposure to palatable hypercaloric diets, rich in lipids, carbohydrates and salt, and pro-obesogenic. In addition, low physical activity has led to an increase in life-threating sedentarism, favoring malnutrition, obesity development and metabolic diseases such as diabetes, dyslipidemia and hypertension (Wanderley and Ferreira, 2010; Barnes, 2011). At the pathological level, overweight (25 kg/m^2^ > BMI < 30 kg/m^2^), contribute at least 2.8 million deaths each year (World Health Organization [WHO], 2021), and negatively affect children and adolescents by increasing the risk of chronic non-communicable diseases (NCDs), such as coronary heart disease, metabolic syndrome, and diabetes in adulthood (Bibbins-Domingo et al., 2007). All this epidemiological data, plus the high availability of processed foods in developed countries, favoring a hedonic feeding behavior and neurophysiological signals that regulate satiation and hunger (Berthoud and Munzberg, 2011; Egecioglu et al., 2011; Lee and Dixon, 2017). In this context, understanding how obesogenic food intake affects brain areas associated with feeding control is a question that has not been fully elucidated.

## Stress and related factors

Stress has been defined as a destabilizing factor of homeostasis, which leads organisms to generate adaptive responses, including the activation of autonomic nervous system and the hypothalamic-pituitary-adrenal (HPA) axis, affecting peripheral organs and brain areas such as the prefrontal cortex (PFC), amygdala, hippocampus and hypothalamus (McEwen and Akil, 2020). In this sense, several investigations have shown that stress causes adaptive plasticity in the brain, in which local neurotransmitters, and systemic hormones, interact to produce structural and functional changes in neural circuit (Patel et al., 2019). However, when demands related to chronic stress become constant, the body adapts to these new demands and these new changes can lead to pathophysiological conditions in other systems, such as depression (Van Praag, 2005; Patel et al., 2019), cardiovascular diseases (Steptoe and Kivimaki, 2012), eating disorders (e.g., bulimia and anorexia nervosa; Lo Sauro et al., 2008; Groesz et al., 2012) and obesity (Patterson and Abizaid, 2013).

Neurobiologically, the HPA axis integrates the physiological response to stress. A stressful stimulus, regardless in nature stimulates parvocellular neurons in the paraventricular nucleus of the hypothalamus (PVN; Smith and Vale, 2006). These neurons release corticotrophin releasing factor (CRF) and arginine vasopressin (AVP) on the pituitary stimulating specific receptors (GPCRs) expressed on corticotropic cells to synthesize and release adrenocorticotropic hormone (ACTH) into the systemic circulation. Then, ACTH activates melanocortin-2 receptor in the adrenal cortex, stimulating the steroidogenesis of glucocorticoids (Smith and Vale, 2006). Glucocorticoids are the main endocrine effectors of the HPA axis, and their levels are highly regulated not only by feedback inhibition at the pituitary and hypothalamus, but also at the hippocampus (Hipp), nucleus of the solitary tract, and pre-limbic cortex (Sapolsky et al., 1984; Blanchard et al., 1993; McEwen and Akil, 2020).

## Stress and food intake

Stress is enough to initiate a highly catabolic physiological response, mobilizing stored energy, thus affecting anabolic processes such as feeding, thirst, and reproduction (Michel et al., 2003; Wingfield and Sapolsky, 2003). However, it is not known whether, the metabolic stress induced by obesogenic diets, nor the chronic stress response might contribute to initiate or reinforce hyperphagic behaviors in obese individuals, showing that CRF system is involved in the regulation of energy balance and the pathophysiology of obesity (Sominsky and Spencer, 2014). CRF was discovered in 1981 as part of the endocrine pathway that regulates ACTH and glucocorticoids (Vale et al., 1981). The CRF system includes CRF and urocortins (UCN; 1, 2, and 3) such as endogenous ligands, CRF receptors type 1 (CRF_1_) and type 2 (CRF_2_), and CRF binding protein (CRF-BP; for revision see Slater et al., 2016b). The affinity of CRF and UCN1 for the CRF_1_ and CRF_2_ are similar, while the UCN2 affinity for CRF_2_ is approximately 5,000 times greater than for CRF_1_. UCN2 and UCN3 are mainly considered selective agonist for CRF_2_ (Dautzenberg et al., 2019).

CRF receptors are widely expressed in other brain regions and peripheral organs, and therefore, CRF has extrahypothalamic effects, beyond HPA axis regulation (for revision see Deussing and Chen, 2018). For instance, it has been shown that systemic and central administration of CRF or UCN significantly decreases food intake, which could be reversed with the administration of a CRF_2_ antagonist, but not with the administration of a CRF_1_ antagonist (Smagin et al., 1998). In this context, administration of ASV-30, a selective CRF_2_ antagonist, to rats with diet-induced obesity has been shown to reduce the anorexigenic effects of CRF or UCN (Cullen et al., 2001). CRF and UCN are endogenous ligands of CRF receptors, so part of the decrease in food intake may be due to the associated activation of the HPA axis and elevation of plasma corticosteroids. On the other hand, it has been shown that UCN3, a selective agonist of CRF_2_, regulates feeding behaviors (for revision see Zhang et al., 2019). For example, both intracerebroventricular administration of UCN3 has been shown to reduce food intake in mice and rats (Pelleymounter et al., 2004; Chen et al., 2010). Interestingly, the hypothalamic expressions of UCN3 and CRF_2_, in lean rats, are high and low in *ad libitum* and fasted feeding condition, respectively (Poulin et al., 2012). However, the UCN3 and CRF_2_ expression levels are constant in obese rats (Poulin et al., 2012). Possibly this and other differences in CRF system could be participating in the maintenance of the obesity condition, especially under conditions of physiological stress such as chronic fasting.

In fact, in patients with anorexia nervosa, characterized by a significant decrease in food intake, the CRF and corticosteroids levels are elevated in plasma (Gross et al., 1994). CRF has been described as a potent anorexigenic agent and, therefore, increasing its bioavailability inhibits food intake. However, the end product of the HPA axis, corticosteroids, triggers orexigenic-type responses, as it influences eating behavior that might lead to excess malnutrition, and therefore obesity (Dallman et al., 2007). In addition, intraventricular injection of glucocorticoids in adrenalectomized (ADX) rats stimulates the CRF system, increasing caloric intake, especially carbohydrate consumption (Kumar and Leibowitz, 1988), while administration of corticosteroids has been shown to increase the intake of highly palatable foods (Bell et al., 2000). In Zucker rats (Duclos et al., 2005) and ob/ob mice, both genetic models of obesity, an increase in plasma levels of corticosteroids has been observed (Makimura et al., 2000; Duclos et al., 2005). While ADX in ob/ob mice normalized food intake (Makimura et al., 2000). In addition, the protracted exposure to a highly-palatable diet was associated with the development of stress-related behaviors, improving when the highly-palatable diet is changed back to chow diet (Sharma et al., 2013). However, the motivation toward palatable rewards was not normalized, associating with a decrease and increase in the expression of CRF_1_ and brain-derived neurotrophic factor (BDNF) in the nucleus accumbens (NAcc), respectively (Sharma et al., 2013). Interestingly, it has been shown that stress-induced CRF system modulate the activity of dopaminergic neurons, affecting the dopamine (DA) release (a neurotransmitter associated with pleasure) in limbic areas such as NAcc and lateral septum (LS; Sotomayor-Zarate et al., 2013; Sotomayor-Zarate et al., 2015; Slater et al., 2016a). In summary, the evidence suggests that the CRF system is related to energy homeostasis, and any alteration in this system will impact food intake behavior.

## The lateral septum

The LS is a structure that is interconnected with brain areas involved to cognition, motivation, autonomic regulation, stress and feeding (Gray, 1977; Sheehan et al., 2004; Patel, 2022). It is part of a subcortical structure that delimits the midline of the brain called “septum” and is divided into two parts: the septum pellucidum and septum verum (Andy and Stephan, 1968). The septum verum is divided into medial (MS) and lateral (LS) parts, which differ in their connectivity and functions (Swanson and Cowan, 1979). The MS is cellularly heterogeneous, being composed mainly of excitatory glutamatergic and modulatory cholinergic neurons (Brashear et al., 1986) with bidirectional connections to the Hipp, a key structure involved in learning, memory, and mood regulation (Raisman, 1966; Takeuchi et al., 2021). The LS is constituted predominantly of GABAergic neurons and interneurons, mainly receiving glutamatergic input from the Hipp and projecting GABAergic output to the lateral hypothalamus (LH), between other brain connections (Risold and Swanson, 1997a,b).

**FIGURE 1:**
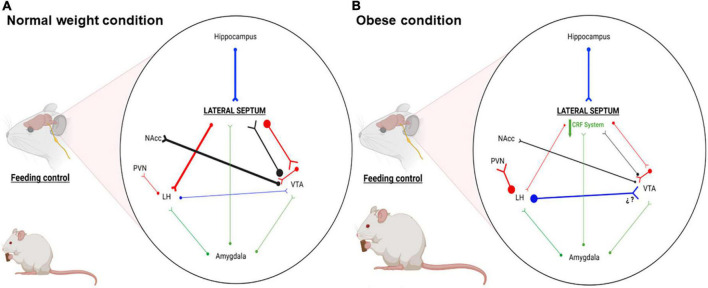
Schematic representation of the main LS connections associated with motivation, stress, emotional and feeding behaviors. **(A)** Representative connectivity in normal weight condition: LS GABAergic efferents regulate activity of LH and VTA neurons, impacting behaviors such as hunger and motivation. **(B)** Representative connectivity in obese condition: Our proposal shows that LS CRF system is downregulated, reducing the activity of LS GABAergic efferents. This reduced activity favors hyperactivation and hypoactivation of LH and VTA, leading to hyperphagia and less reward for foods. Blue, glutamatergic neurons; Red, GABAergic neurons; Black, dopaminergic neurons; Green, CRFergic neurons; NAcc, nucleus accumbens; LH, lateral hypothalamus; PVN, paraventricular nucleus; VTA, ventral tegmental area. This figure was created with BioRender.com under subscription and have a license of Biorender to use the figure in journal publications.

Anatomically, LS is located in the subcortical forebrain, between the lateral ventricles, slightly dorsorostral to the nucleus accumbens (NAcc), dorsocaudal to the hypothalamus and ventral to corpus callosum (Swanson and Cowan, 1979). LS has been considered a neural relay station in the forebrain, related to the control of motivation, anger, stress and autonomic regulation (for revision see Sheehan et al., 2004; Figure 1). Also, LS integrates emotions, localization, cognitive information and motivation by rewarding stimuli (Rizzi-Wise and Wang, 2021). The pioneering work of Olds and Milner showed that LS is able to support self-stimulation suggesting its role on the reward system (Olds and Milner, 1954). In addition, Brady and Nauta (1953) suggest that the septum plays a prominent role in controlling stress-induced behavior. The role of LS in addiction has been poorly explored. Sotomayor et al. showed that systemic morphine administration increases LS DA extracellular levels through a decrease in GABA levels in ventral tegmental area (VTA; Sotomayor et al., 2005). In addition, LS GABAergic neurons activate the firing of VTA DA neurons, through the inhibition of VTA GABA interneurons (Luo et al., 2011; Vega-Quiroga et al., 2018; Figure 1). Finally, LS GABA neurons express receptors for several neurotransmitters and neuropeptides that modulate it neural activity, such as vasopressin (Garate-Perez et al., 2021), norepinephrine (NE; Scopinho et al., 2008), ghrelin (Terrill et al., 2018), glucagon-like peptide 1 (GLP-1; Terrill et al., 2016) and CRF (Sotomayor-Zarate et al., 2013), between others.

## Lateral septum and feeding

Despite the inhibitory role of LS on LH (an orexinergic nucleus involved in homeostatic control of feeding), the role of LS in feeding has been poorly studied. Pankey et al. found opposing results on septal lesions and body weight. Lesion of the septal area in male rats produced weight loss, while in female rats induced weight gain (Pankey et al., 2008). On the other hand, the noradrenergic system in the LS is involved in feeding behavior. The LS microinjection of NE increases food intake and this effect is blocked when it is co-microinjected an α1-adrenergic receptor antagonist (Scopinho et al., 2008). LS is strongly activated by glutamatergic input from the cornus ammonis area 3 (CA3) of ventral hippocampus (vHipp), increasing LH GABA release from LS projection (for revision see Rizzi-Wise and Wang, 2021). It should be noted that LH activation triggers hunger behavior through intrahypothalamic GABAergic projections from LH to PVN, where anorexigenic neurons are inhibited (Boudaba et al., 1996). In this context, the LS activation would inhibit the activity of LH neurons and the associated behaviors. The importance of the Hipp-LS-LH pathway in the feeding control has been recently tested using Designer Receptors Exclusively Activated by Designer Drugs (DREADD)-based chemogenetic tools. One hand, the chemogenetic activation expressing hM3Dq in CA3-vHipp glutamatergic efferents to LS reduce food intake (Sweeney and Yang, 2015) and the chemogenetic activation (expressing hM3Dq) or inhibition (expressing hM4Di) of LS GABAergic neurons produce a reduction and increase in food intake, respectively (Sweeney and Yang, 2016). On the other hand, the chemogenetic activation (expressing hM3Dq) in LS neurotensin neurons that project to LH decreases food intake (Azevedo et al., 2020). Recently, it has been shown that the activation of LS neurotensin neurons that project to the *nucleus tuberalis lateralis*, a nucleus located posterior part of the hypothalamus, reduce the intake of palatable foods (rich in fat and sucrose) and sweet solutions (sucrose and Ensure) in *ad libitum* and fasted condition (Chen et al., 2022).

Regard to neuropeptide systems that could regulate the activity of LS neurons, it has been shown that LS microinjection of GLP-1 decreases control food intake, while LS microinjections of antagonists of GLP-1 receptors only increases palatable food intake (Terrill et al., 2019). Recently, it has been shown that acute restraint stress increases the activity of LS neurons that express the GLP-1 receptor, decreasing food intake in male mice fed a control diet (Bales et al., 2022). However, in obese male mice the restraint stress does not increase the activity of LS GLP-1R neurons or produce hypophagia (Bales et al., 2022). Finally, the LS microinjection of μ-receptor agonists such as morphine and DAMGO reduces and increases the latency to eat and food intake, respectively (Calderwood et al., 2022).

## The lateral septum - corticotropin releasing factor system on feeding

CRF_1_ mRNA expression has been found predominantly in the forebrain, olfactory regions, cerebellum, anterior pituitary corticotroph cells and in the PVN parvocellular zone (Bravo et al., 2011). On the other hand, CRF_2_ is expressed in the anterior hypothalamic area (AHA), ventromedial hypothalamus (VMH), nucleus tractus solitarius (NTS), dorsal raphe nucleus, and LS (Van Pett et al., 2000; Deussing and Chen, 2018). In this context, LS is a brain nucleus with high density of CRF, UCN1, and UCN3 containing nerves fibers from hypothalamic (PVN and supraoptic nuclei) and extra-hypothalamic areas (Edinger-Westphal), respectively (Joseph and Knigge, 1983; Swanson et al., 1983; Bittencourt et al., 1999).

At neurophysiological level, LS is considered as a brain relay station that plays an important role in regulate the limbic system, interpretation of several sensory inputs involved in stress, anxiety, motivation and homeostasis, between others (Calhoon and Tye, 2015). Furthermore, different types of stressors such as food deprivation, predator odor, subordination stress, exposure to aversive sounds, and chronic exposure to drugs of abuse, significantly activate the LS (Martin and Timofeeva, 2010; Mirrione et al., 2014). LS CRF receptors promote plasticity to exogenous stimuli such as chronic exposure to drugs of abuse. In this context, electrophysiological studies show that LS CRF_2_ activation decreases postsynaptic excitatory currents in rats chronically exposed to cocaine (Liu et al., 2004). In addition, *in vivo* optogenetic activation and inhibition of LS CRF_2_ evoked and suppressed anxiety-like behaviors, respectively (Anthony et al., 2014). On the other hand, chronic unpredictable stress (14 days) increases LS CRF_2_ mRNA (Malta et al., 2021).

Regard to food intake, LS UCN infusions produce a reduction in food intake, that it is prevented with the infusions of Astressin-2B, a potent and selective CRF_2_ antagonist (Bakshi et al., 2007). Acute stress exposure has been associated with anorexigenic and anhedonic responses to food (Foster et al., 2009), while chronic stress exposure increases or decreases eating behavior, generating under- and overeating phenotypes (Calvez and Timofeeva, 2016). However, chronic exposure to hypercaloric diets and stressors promote the development of overeating disorders in humans and binge eating behaviors in rodents (Mathes et al., 2009). Highly palatable foods have a positive valence that promotes a positive emotional state, decreasing negative emotions induced by stress and anxiety (Blasio et al., 2013). These effects trigger maladaptive behaviors to seek and eat palatable foods, promoting a positive feedback loop to alleviate chronic stress and favor overweight and obesity (Ulrich-Lai, 2016). In addition, the exposure to chronic stress stimulates the expression of relaxin-3, an orexinergic neuropeptide, that contribute to amplifying the palatable food intake (Calvez and Timofeeva, 2016; Calvez et al., 2017). In this sense, LS presents a high expression of CRF receptors, receives inputs from CRFergic neurons and it has an important role in regulating the neural activities in LH and VTA, which make it a good candidate to study feeding processes in stress conditions.

## Summary and conclusion

The LS has been implicated in the regulation of motivation and feeding. This nucleus expresses several receptors for neurotransmitters and neuropeptides involved in feeding control, including CRF, neuropeptide Y, ghrelin, β-endorphin and GLP-1, between others. The main GABAergic projection of the LS goes to the LH, inhibiting it and reducing food intake. Furthermore, others LS GABAergic projections go to the VTA, increasing the firing of DA neurons. The septal connection shows us an integrative role of this nucleus on homeostatic and hedonic control feeding. However, the regulatory role of the LS on food intake has not been evaluated in obesity, so evaluating whether the lateral septum has a role in the pathophysiology of obesity or whether it can be considered a therapeutic target to treat this disease, are focuses of interest for our research group.

## Author contributions

RO-B, JM, and RS-Z wrote the manuscript. JM-P, MF, KG, JB, and RS-Z revised and edited the manuscript. RS-Z obtained the funding. All authors approved the final version of the manuscript.
